# Sleep quality mediates the association between tea consumption and duration of COVID-19-related symptoms in middle-aged and elderly adults (aged 50 and above)

**DOI:** 10.3389/fpsyt.2025.1603257

**Published:** 2025-08-13

**Authors:** Yuxin Fan, Yaonan Zhu, Yunyu Wang, Jun Jiang, Shaopeng Yang, Jie Lu, Qinghua Ma, Hong Zhu

**Affiliations:** ^1^ School of Public Health, Medical College of Soochow University, Suzhou, China; ^2^ Department of Preventive Health Care, The 3rd People's Hospital of Xiangcheng District, Suzhou, China; ^3^ Yuanhe Street Community Health Service Center, Xiangcheng District Dermatosis Hospital, Suzhou, China; ^4^ School of Public Health, North China University of Science and Technology, Tangshan, China; ^5^ School of Public Health and Health Management, Anhui Medical College, Hefei, China; ^6^ Center for Disease Control and Prevention, Health Commission of Zhanhua District, Binzhou, China

**Keywords:** tea consumption, sleep quality, duration of COVID-19-related symptoms, mediation analysis, middle-aged and elderly adults

## Abstract

**Objectives:**

The association between tea consumption and the duration of COVID-19-related symptoms remains inconclusive. This cross-sectional study investigates the potential mediating role of sleep quality in this association. The association between tea consumption and the duration of COVID-19-related symptoms remains inconclusive. This cross-sectional study aims to investigate the potential mediating role of sleep quality in this association.

**Methods:**

We conducted a cross-sectional study using data from middle-aged and elderly adults (aged 50 and above) in Weitang Town in 2023. Detailed information on tea consumption, duration of COVID-19-related symptoms, and sleep quality was collected through face-to-face interviews using pre-designed questionnaires. Sleep quality was assessed using the Pittsburgh Sleep Quality Index (PSQI), which evaluated sleep quality over the past month during the acute phase of COVID-19. Spearman correlation analysis was employed to examine the relationships between variables. mediation analysis utilized a mediation model with multi-category independent variables.

**Results:**

Tea consumption was negatively associated with sleep quality, which in turn was positively associated with COVID-19 symptom duration. Mediation analysis showed sleep quality partially mediated the relationship between daily tea drinking and symptom duration, and fully mediated associations between green tea consumption, tea drinking for <15 or ≥30 years, tea concentration, and symptom duration. The mediation effect accounted for 11%–21% of the total effect.

**Conclusions:**

Tea consumption is associated with shorter duration of COVID-19-related symptoms, with sleep quality acting as a mediator. These findings highlight the potential of improving sleep quality to reduce symptom duration, but conclusions are limited by the cross-sectional design.

## 1 Introduction

The COVID-19 pandemic has presented an unprecedented public health crisis, with over 770 million reported cases worldwide as of November 2023 (1). While most infected individuals experience mild symptoms (2), some symptoms can persist for an extended period (3). COVID-19 symptom duration varies widely among individuals, with factors such as immune function and lifestyle habits contributing to these differences (4, 5). Specifically, prolonged symptom duration has been linked to poor sleep quality and inadequate dietary patterns (6). Common symptoms include fever, nasal congestion, sore throat, hoarse voice, muscle pain, headache, among others. Research has indicated that long-term clinical manifestations following the acute phase of the illness can result in chronic inflammation and immune dysregulation (7, 8).

Tea is widely enjoyed and has gained considerable attention. As a natural food, tea has been utilized in ancient China for treating certain infectious diseases. Previous studies have directly associated tea consumption with shorter symptom duration in viral infections, potentially via modulation of immune responses (9). In modern times, drinking tea offers numerous health benefits, including the prevention of type 2 diabetes, obesity, cardiovascular disease, cancer, and immune-related diseases. These benefits are attributed to tea’s anti-inflammatory, immunomodulatory, and antioxidant properties (10). Tea was selected as the focus due to its cultural significance in Suzhou, where local residents have a long history of tea consumption (11). In this region, tea is a staple beverage, making it a relevant exposure variable for the study population.

Additionally, tea can promote sleep by calming the nerves (12). Tea’s effects on sleep are complex: caffeine may disrupt sleep, while theanine promotes relaxation, which has led to conflicting findings in the literature (13). Sleep quality is crucial for maintaining a robust immune system, as poor sleep quality increases susceptibility to viral infections (14). Studies have indicated that COVID-19 patients with inadequate sleep experience prolonged duration of symptoms (15). Additionally, lifestyle factors have been shown to mediate infectious disease outcomes, though few studies have explored this in the context of COVID-19 (16).

Both tea consumption and sleep quality have an impact on the duration of COVID-19-related symptoms, and they mutually influence each other. However, the underlying mechanism remains unclear. In our study, we hypothesized that individuals who consume tea would experience a reduced duration of COVID-19-related symptoms, with sleep quality playing a mediating role. Therefore, this project aims to investigate the relationship between tea consumption, sleep quality, and the duration of COVID-19-related symptoms.

## 2 Materials and methods

### 2.1 Study subjects

Participants were adults aged 50–90 from Weitang Town, Suzhou, recruited via community health centers. Exclusion criteria: incomplete data or inability to complete interviews. A total of 2395 participants were included. Participants were non-hospitalized individuals confirmed to have COVID-19 via nucleic acid testing, all of whom had sought medical attention due to COVID-19-related symptoms (e.g., fever, cough, sore throat) during the acute phase of infection. Data were collected through face-to-face interviews using pre-designed questionnaires administered by trained researchers. All provided written informed consent. Ethical approval was obtained from the Third People’s Hospital of Xiangcheng District (xcsyllpj2023-001).

### 2.2 Measures and variables

#### 2.2.1 Basic demographics

Demographic information includes: age, sex, height, body weight, body mass index (BMI).

#### 2.2.2 Tea consumption

Tea consumption was assessed based on the frequency, type, duration, and tea concentration. Frequency of tea consumption was categorized as daily (≥1 cup/day), occasional (≥1 cup/week or ≥1 cup/month), rarely (<1 cup/month), or never, which is consistent with the tea consumption pattern classifications found in relevant population study (17). The types of tea consumed were classified as green tea or non-green tea. Duration of tea consumption was categorized as <15 years, 15–30 years, and ≥30 years. Tea concentration was determined by the proportion of tea leaves in the cup after brewing, with <25% considered light tea, 25% to 50% considered medium strength tea, and >50% considered strong tea. This classification is in line with international studies on tea consumption behaviors (18).

#### 2.2.3 Sleep quality

The Pittsburgh Sleep Quality Index (PSQI) is employed to evaluate sleep quality, consisting of seven components: subjective sleep quality, sleep latency, sleep duration, habitual sleep efficiency, sleep disturbances, use of sleep medication, and daytime dysfunction. Total PSQI scores were calculated by summing the scores of these seven components. Scores can range from 0 to 21, with higher scores indicating poorer sleep quality (19). The PSQI assessed sleep quality during the month prior to COVID-19 infection to capture pre-infection sleep patterns.

#### 2.2.4 Duration of COVID-19-related symptoms

The duration (in days) of COVID-19-related symptoms refers to the period when symptoms occur following infection with COVID-19 during the pandemic. These symptoms encompass fever, nasal congestion, sore throat, hoarse voice, muscle pain, headache, cough, difficulty breathing, heart palpitations, vomiting, skin rashes, changes in vision, altered smell, loss of smell, and diarrhea.

### 2.3 Statistical analyses

The data were analyzed using IBM SPSS Statistics 27.0. Normally distributed continuous variables are presented as mean ± standard deviation, non-normally distributed continuous variables are reported as median (interquartile range), and categorical variables are presented as counts and percentages [n (%)]. Differences between males and females were assessed using t-tests, chi-square tests, and rank sum tests. The Spearman correlation method was employed to examine the relationships between tea consumption frequency, type, duration, concentration, sleep quality, and duration of COVID-19-related symptoms.

Mediation analysis was conducted using Model 4 of PROCESS with 5000 bootstrap samples, developed by Hayes, with Bootstrapping (20). Mediation models adjusted for covariates including age, gender, and BMI, as these factors are known to influence sleep quality and COVID-19 symptom duration (21). Partial mediation was defined as a significant indirect effect with a remaining significant direct effect, while full mediation required a significant indirect effect and non-significant direct effect. All tests were two-tailed, and statistical significance was set at p <0.05.

## 3 Results

### 3.1 Basic information about the research object

The participants had an mean age of 67.68 ± 0.29 years (41.2% male). The participants had a mean BMI of 23.57 ± 0.06 kg/m^2^. The average sleep quality score was 4.34 ± 0.07. The median duration of COVID-19-related symptoms was 4.00 (1.00, 7.00) days. Male participants were significantly older than female participants (males: 70.32 ± 0.17 years; females: 65.82 ± 0.21 years; *p*<0.05). There were no significant differences, except for BMI, between male and female subgroups. Significant differences in tea consumption, sleep quality, and duration of COVID-19-related symptoms were observed between these two subgroups (**Table 1**).

**Table 1:** Participant characteristics (n=2395).

Variables	Total	Male	Female	*P*-value
Number	2395 (100)	987 (41.2)	1408 (58.7)	
Age (years)	67.68 ± 0.29	70.32 ± 0.17	65.82 ± 0.21	<0.05
Body mass index	23.57 ± 0.06	23.50 ± 0.10	23.62 ± 0.09	0.35
Frequency of tea consumption				<0.05
rarely or never	69.65	42.86	88.42	
occasional tea consumption	5.09	6.08	4.40	
daily tea consumption	25.26	51.06	7.17	
Type of tea consumption				<0.05
non-green tea	73.44	47.52	91.62	
green tea	26.56	52.48	8.38	
Duration of tea consumption				<0.05
non-tea	69.65	42.86	88.42	
<15 years	6.10	6.79	5.61	
15–30 years	7.10	11.85	3.76	
≥30 years	17.16	38.50	2.20	
Tea concentration				<0.05
non-tea	69.65	42.86	88.42	
light tea	16.28	27.76	8.24	
medium strength tea	7.77	15.81	2.13	
strong tea	6.30	13.58	13.58	
PSQI score	4.34 ± 0.07	3.62 ± 0.09	4.85 ± 0.10	<0.05
Symptom duration (days)	4.00 (1.00,7.00)	3.00 (0.00,7.00)	6.00 (3.00,8.00)	<0.05

### 3.2 Correlation analysis

A Spearman correlation analysis was conducted to examine the relationship between tea consumption, sleep quality, and duration of COVID-19-related symptoms (**Table 2**). The results indicated that tea consumption frequency, type, duration, and concentration were negatively correlated with sleep quality (PSQI-assessed) (*r*=-0.098 to -0.107, *p* < 0.01). This suggests that higher frequency of tea consumption, consumption of green tea, longer duration of tea consumption, and higher tea concentration are associated with better sleep quality. Additionally, PSQI scores were positively correlated with the duration of COVID-19-related symptoms (*r* = 0.204, *p* < 0.001), indicating that poor sleep quality is linked to a longer duration of COVID-19-related symptoms. Moreover, there was a negative correlation between tea consumption frequency, type, duration, concentration, and the duration of COVID-19-related symptoms (*r*=-0.144 to -0.150, *p* < 0.01). In other words, higher tea consumption frequency, consumption of green tea, longer duration of tea consumption, and higher tea concentration were associated with a shorter duration of COVID-19-related symptoms.

**Table 2:** Spearman correlation analysis among perceived tea consumption, sleep quality, and duration of COVID-19-related symptoms.

Variables	Frequency of tea consumption	Type of tea consumption	Duration of tea consumption	Tea concentration	Sleep quality	Duration (days) of COVID-19-related symptoms
Frequency of tea consumption	—					
Type of tea consumption	0.907^**^	—				
Duration of tea consumption	0.982^**^	0.908^**^	—			
Tea concentration	0.979^**^	0.895^**^	0.972^**^	—		
Sleep quality	-0.107^**^	-0.105^**^	-0.102^**^	-0.098^**^	—	
Duration(days) of COVID-19-related symptoms	-0.145^**^	-0.145^**^	-0.150^**^	-0.144^**^	0.204^**^	—

**p<0.01.

### 3.3 Results of mediation analysis

#### 3.3.1 The mediating role of sleep quality between frequency of tea consumption and duration of COVID-19-related symptoms

**Figure 1:**
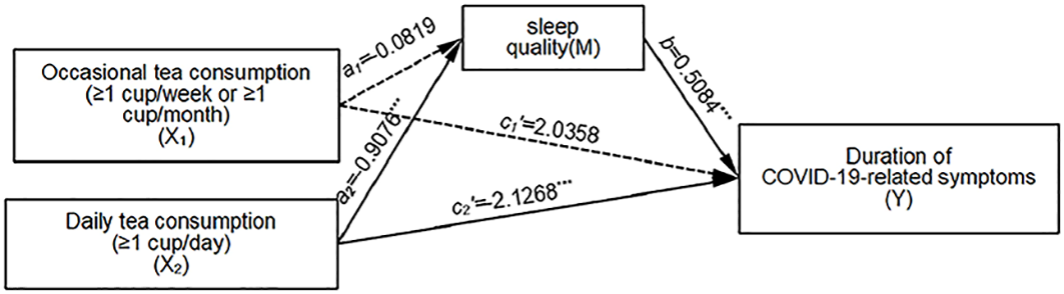
Mediation Model of Sleep Quality Between Tea Drinking Frequency and COVID-19 Symptom Duration. Unstandardized coefficients shown; solid lines indicate significant paths (***p < 0.001), dotted lines indicate non-significant paths. X_2_ vs. non-drinkers: *a_2_* = -0.9076 (daily tea → sleep quality, *p* < 0.001). *b* = 0.5084 (sleep quality → symptom duration, *p* < 0.001). *c_2_’* = -2.1268 (direct effect, *p* = 0.0195). Indirect effect: a_2_b = -0.4614 (95% CI [-0.7478, -0.2343]). Total effect: c_2_ = a_2_b + c_2_’ = -2.5882 (mediation proportion: 17.8%). Interpretation:Sleep quality partially mediates the association between daily tea drinking and symptom duration, as direct effect remains significant.

Relative mediation analysis revealed a significant relative mediation effect when comparing daily tea drinkers to non-tea drinkers, with a 95% confidence interval of [-0.7478, -0.2343]. This indicates that daily tea consumption is associated with higher sleep quality and a reduced duration of COVID-19-related symptoms (*a_2_* =-0.9076, *b*=0.5084, *a_2_b*=-0.4614). Moreover, the relative direct effect was significant (*c_2_’*=-2.1268, *p*<0.001), indicating that daily tea drinkers have a shorter duration of COVID-19-related symptoms compared to non-tea drinkers when the mediating effect is excluded. The relative total effect was also significant (*c_2_* =*a_2_b*+*c_2_’*=-2.5882, *p*<0.001), with the relative mediation effect accounting for 17.8% of the total effect size (0.4614/2.5882). Conversely, when comparing occasional tea drinkers to non-tea drinkers, the 95% confidence interval was [-0.3724, 0.3053], indicating no significant relative mediation effect (*a_1_* =-0.0819, *b*=0.5084, *a_1_b*=-0.0417). Detailed information is provided in **Figure 1** and **Table 3**.

**Table 3:** Multicategory mediation modeling: mediating effects of sleep quality in the influence of occasional and daily tea consumption on the duration of COVID-19-related symptoms, n=2395.

Trails	Unstandardized coefficients (S.E.)	*P*	95% confidence interval
Sleep quality			
Occasional tea consumption (*a_1_*)	-0.0819 (0.3170)	0.7961	-0.7036,0.5397
Daily tea consumption (*a_2_*)	-0.9076 (0.1604)	<0.001	-1.2222,-0.5931
Duration of COVID-19-related symptoms			
sleep quality (*b*)	0.5084 (0.1152)	<0.001	0.2825,0.7342
Occasional tea consumption (*c_1_’*)	2.0358 (1.7860)	0.2545	-1.4665,5.5380
Daily tea consumption (*c_2_’*)	-2.1268 (0.9098)	0.0195	-3.9108,-0.3427
Relative indirect effects			
*a_1_b*	-0.0417 (0.1660)	—	-0.3724,0.3053
*a_2_b*	-0.4614 (0.1302)	—	-0.7478,-0.2343
Relative total effects			
*a_1_b*+*c_1_’*	1.9941 (1.7929)	0.2661	-1.5216,5.5098
*a_2_b*+*c_2_’*	-2.5882 (0.9072)	0.0044	-4.3672,-0.8091

Relative indirect effects (ab) represent standardized mediation effect sizes.

#### 3.3.2 The mediating role of sleep quality between type of tea consumption and duration of COVID-19-related symptoms

**Figure 2:**
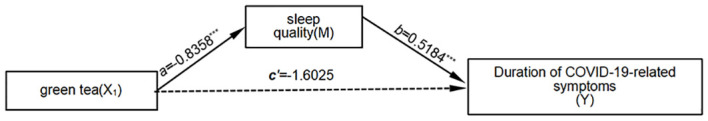
Mediation Model of Sleep Quality Between Tea Type and COVID-19 Symptom Duration. Unstandardized coefficients shown; solid lines indicate significant paths (***p < 0.001), and dotted lines denote non-significant paths. Key Coefficients. *a* = -0.8358 (green tea → sleep quality, *p*< 0.001). *b*= 0.5184 (sleep quality → symptom duration, *p* < 0.001) *c’* = -1.6025 (direct effect, *p* = 0.0709). Indirect effect: ab = -0.4333 (95% CI [-0.7071, -0.2181]). Mediation proportion: 21.3%. Interpretation:Green tea’s association with shorter symptom duration is fully mediated by sleep quality.

We utilized Model 4 in PROCESS to examine the impact of sleep quality on the relationship between tea consumption type and the duration of COVID-19-related symptoms. The findings revealed that the direct prediction of the duration of COVID-19-related symptoms by tea drinking type was not significant (*c’*=-1.6025, *p*=0.0709). When comparing green tea drinkers to non-drinkers, with non-drinking of green tea as the reference level, the 95% confidence interval was [-0.7071, -0.2181], indicating a significant mediation effect (*a*=-0.8358, *b*=0.5184, *ab*=-0.4333). This implies that individuals who consume green tea have higher sleep quality and experience a reduced duration of COVID-19-related symptoms. The direct effects were found to be non-significant, suggesting that sleep quality exhibited full mediation in the model. Moreover, the total effect was significant (*c*=*ab*+*c’*=-2.0358, *p*<0.001), with the mediation effect accounting for 21.3% of the total effect size (0.4333/2.0358). For detailed information, refer to **Figure 2**.

#### 3.3.3 The mediating role of sleep quality between tea consumption duration and COVID-19-related symptoms

**Figure 3:**
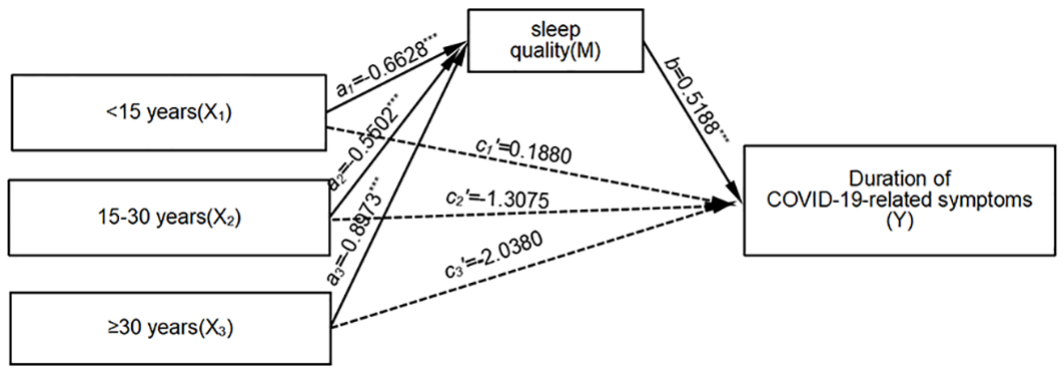
Mediation Model of Sleep Quality Between Tea Consumption Duration and COVID-19 Symptom Duration. Unstandardized coefficients are shown. Solid lines denote significant paths (***p < 0.001), and dotted lines denote non-significant paths. X_3_ vs. non-drinkers: *a_3_* = -0.8973 (≥30 years → sleep quality, *p* < 0.001). *b*= 0.5188 (sleep quality → symptom duration, *p* < 0.001). *c_3_’* = -2.0380 (direct effect, *p* = 0.123). Indirect effect: a_3_b = -0.4656 (95% CI [-0.7821, -0.2491]). Mediation proportion: 18.6%. Interpretation:Long-term tea consumption (≥30 years) influences symptom duration fully through sleep quality.

Relative mediation analysis revealed a significant mediating effect when comparing tea drinking for less than 15 years and tea drinking for more than 30 years to non-tea drinking (*a_1_* =-0.6628, *b*=0.5188, *a_1_b*=-0.3439; *a_3_* =-0.8973, *b*=0.5188, *a_3_b*=-0.4656). This indicates that individuals who have been consuming tea for less than 15 years and those who have been consuming tea for more than 30 years exhibit better sleep quality compared to non-tea drinkers. Moreover, both groups experience a corresponding reduction in COVID-19-related symptoms. The relative direct effects are not significant, suggesting that sleep quality exhibited full mediation in the model. The relative total effect of consuming tea for less than 15 years, compared to non-tea drinking, is not significant. However, the relative total effect of individuals who have been consuming tea for more than 30 years, compared to non-tea drinking, is significant (*c_3_* =*a_3_b*+*c_3_’*=-2.5036, *p*<0.001). The effect size of the relative mediation effect is 18.6% (0.4656/2.5036). It is important to note that sleep quality does not mediate the relationship between tea drinking for 15–30 years and COVID-19-related symptoms. For further details, refer to **Figure 3**.

#### 3.3.4 The mediating role of sleep quality between tea concentration and duration of COVID-19-related symptoms

**Figure 4:**
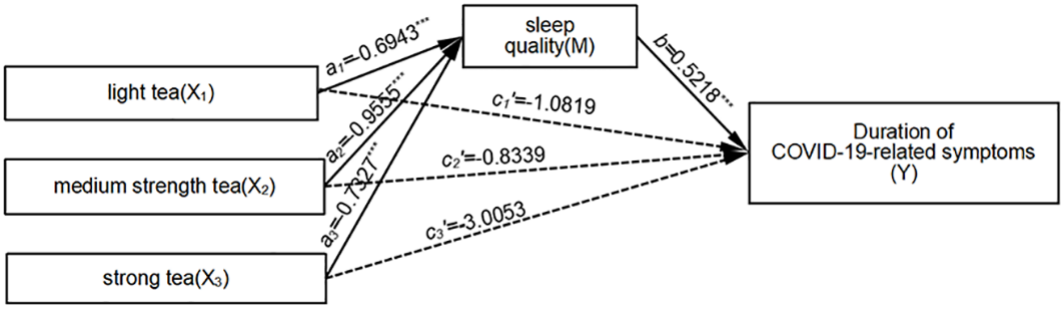
Mediation Model of Sleep Quality Between Tea Concentration and COVID-19 Symptom Duration. Unstandardized coefficients are shown. Solid lines denote significant paths (***p < 0.001), and dotted lines denote non-significant paths. a_3_ = -0.7327 (strong tea → sleep quality, *p* < 0.001). *b* = 0.5188 (sleep quality → symptom duration, *p* < 0.001). c_3_’ = -3.3876 (direct effect, p < 0.001). Indirect effect: a_3_b = -0.3823 (95% CI [-0.6742, -0.0904]). Mediation proportion: 11.3%. Interpretation:Strong tea consumption influences symptom duration fully through sleep quality.

Relative mediation analysis revealed a significant mediating effect when comparing drinking light tea, moderate tea, and strong tea to not drinking tea, using non-tea drinking as the reference level (*a_1_* =-0.6943, *b*=0.5218, *a_1_b*=-0.3623; *a_2_* =-0.9555, *b*=0.5218, *a_2_b*=-0.4986; *a_3_* =-0.7327, *b*=0.5188, *a_3_b*=-0.3823). This indicates that individuals who consume light tea, moderate tea, and strong tea experience better sleep quality compared to non-tea drinkers. Additionally, they also exhibit a reduction in COVID-19-related symptoms. The relative direct effects are not significant, suggesting that sleep quality exhibited full mediation in the model. The relative total effect of drinking light tea and moderate tea, compared to not drinking tea, is not significant. However, the relative total effect of drinking strong tea, compared to not drinking tea, is significant (*c_3_* =*a_3_b*+*c_3_’*=-3.3876, *p*<0.001). The effect size of the relative mediation effect is 11.3% (0.3823/3.3876). For further details, refer to **Figure 4**. Overall, mediation effects accounted for 11%-21% of the total effect, indicating a modest mediating role of sleep quality.

## 4 Discussion

The COVID-19 pandemic has emerged as a significant public health crisis, impacting not only my country but also the global community. It has spread rapidly, has affected a wide population, and has posed challenges in terms of prevention and control efforts. This study retrospectively examined cases of Omicron variant infection during the December 2023 pandemic. The findings revealed that the median duration of COVID-19-related symptoms was 4.00 (1.00, 7.00) days, which was shorter compared to the median time of 10.3 (7.4, 12.4) days for nucleic acid tests to turn negative in a makeshift hospital in Shanghai in May 2022 (22). This difference can likely be attributed to the reduced virulence of the virus during the ongoing pandemic, resulting in less severe infections among individuals.

Examination of tea consumption patterns reveals that a relatively small proportion of individuals, approximately 30.35%, consume tea. Among these tea consumers, for the distribution between males and females, it is 57.14% and 11.58%, respectively. Furthermore, male tea drinkers exhibit significantly longer tea drinking durations and higher tea consumption concentrations, aligning with previous research findings (23). The assessment of sleep quality revealed an average score of 4.34 points among participants, with scores ranging from 0 to 5. This score indicates a higher sleep quality compared to the study conducted by Xiaolei Liu et al. in 2022 (PSQI score: 6.16 ± 3.29) (24). In essence, the overall sleep quality is deemed to be very good. These findings corroborate similar studies, consistently indicating that men tend to have significantly better sleep quality than women (25). Gender differences in tea consumption and sleep quality may relate to social drinking habits and hormonal variations (26). For example, males in this study reported higher tea intake, which could be linked to greater social engagement, while estrogen fluctuations in females may contribute to poorer sleep. Consequently, interventions aimed at improving sleep quality should prioritize women and provide additional attention to this demographic.

Notably, despite green tea being culturally popular in Suzhou, only 26.56% of participants consumed it. This discrepancy may reflect the older age of the sample, as middle-aged and elderly adults (aged 50 and above) in Suzhou often prefer non-green teas (27).

In this study, we investigate the relationship between tea drinking and the duration of COVID-19-related symptoms in middle-aged and elderly adults (aged 50 and above). Additionally, we examine the mediating effect of sleep quality on this association. The findings indicate that sleep quality serves as a significant mediator between daily tea consumption, consumption of green tea, duration of tea drinking (less than 15 years and more than 30 years), tea concentration, and the duration of COVID-19-related symptoms.

Tea, a popular beverage rich in phytochemicals, is increasingly recognized for its potential health benefits, including associations with reduced risk of certain diseases. A recent Mendelian randomization study has highlighted causal links between tea intake and improved outcomes in respiratory health, supporting the biological plausibility of tea’s role in modulating immune responses relevant to infectious diseases like COVID-19 (28). Tea may regulate gut microbiota to influence immunity, though this mechanism was not directly measured and requires validation (29). These empirical findings align with the results of our study, which indicate that daily tea consumption is associated with a reduction in the duration of COVID-19-related symptoms.

Tea drinking behavior is associated with sleep quality, which in turn relates to COVID-19-related symptoms. This is attributed to the various health benefits of tea, such as promoting sleep, reducing stress, and regulating energy metabolism (30). However, there are ongoing debates regarding the specific effects of tea type (12, 31, 32), frequency, and quantity on sleep quality. Among the different types of tea, green tea is particularly popular in Suzhou. Green tea is rich in bioactive compounds, including theanine, caffeine, and tea polyphenols. Theanine possesses anti-fatigue, sedative, and sleep-enhancing properties, counteracting the potential negative effects of caffeine on sleep quality (33); Tea polyphenols act as antioxidants, effectively reducing inflammation and oxidative stress caused by sleep deprivation, thereby improving sleep quality and memory (34). However, relevant studies have pointed out that the health effects of tea depend on consumption patterns: while moderate intake may enhance bioactive compound exposure (e.g., polyphenols), excessive consumption—particularly of high-concentration tea—could lead to adverse effects such as caffeine overexposure, which may counteract sleep benefits in sensitive individuals (35). Tea polyphenols act as antioxidants, effectively reducing inflammation and oxidative stress caused by sleep deprivation, thereby improving sleep quality and memory (36). Consistent with these findings, our study demonstrates that individuals who consume green tea exhibit better sleep quality. Furthermore, the duration of tea drinking also plays a significant role, with long-term tea consumption of 30 years or more positively impacting sleep quality. Habitual tea drinking is associated with an improved health-related quality of life in middle-aged and elderly Chinese adults (aged 50 and above), which aligns with our data findings on the relationship between tea drinking and sleep quality (37). Moreover, research suggests that the benefits of tea may be amplified by consuming stronger tea varieties with higher concentrations of beneficial compounds (38).

The duration of COVID-19 symptoms is inversely related to sleep quality. A prospective association exists between sleep disturbance and the development of long-term symptoms after COVID-19. Lower sleep quality, more severe insomnia, and shorter sleep duration 1 to 3 months post-COVID-19 are associated with higher odds of developing a wide range of clinical manifestations. Moreover, studies have reported the efficacy of various tea ingredients in blocking COVID-19 infection (39). Tea polyphenols, for example, can inhibit viruses by positively influencing the gut microbiota. Additionally, increased tea consumption has been associated with a reduced risk of COVID-19 infection (9). Based on the aforementioned research, it can be inferred that tea drinking behavior is associated with better sleep quality, which may relate to reduced impact of COVID-19 symptoms. Therefore, tea consumption not only affects post-COVID-19 symptoms by regulating the internal intestinal flora, as observed in previous studies, but also indirectly influences these symptoms through its impact on sleep.

## 5 Conclusion

This study has several limitations. Firstly, due to the nature of the cross-sectional design, our study was unable to establish a causal relationship between tea consumption, sleep quality, and duration of COVID-19 symptoms. Future research could employ a longitudinal design to address this limitation. Secondly, the information regarding tea drinking, sleep, and COVID-19 was collected through self-reporting methods, which may be subject to retrospective inaccuracies. In future studies, objective measurements such as sleep monitoring watches could be utilized to assess sleep quality. Thirdly, our research subjects were limited to residents of Weitang Town, which exhibits a certain geographical and population concentration. Therefore, a larger sample size may be necessary to generalize the findings to the entire country.

Our study indicates that sleep quality plays a partial mediating role in the relationship between tea consumption and COVID-19-related symptoms among the middle-aged and elderly adults. As a result, our findings not only contribute to a deeper understanding of the mechanism by which tea consumption affects the duration of COVID-19-related symptoms, but also offer new insights for the prevention and treatment of COVID-19 infection.

## Data Availability

The raw data supporting the conclusions of this article will be made available by the authors, without undue reservation.
